# Kinetics of immune responses elicited after three mRNA COVID-19 vaccine doses in predominantly antibody-deficient individuals

**DOI:** 10.1016/j.isci.2022.105455

**Published:** 2022-10-28

**Authors:** Erola Ainsua-Enrich, Núria Pedreño-Lopez, Carmen Bracke, Carlos Ávila-Nieto, María Luisa Rodríguez de la Concepción, Edwards Pradenas, Benjamin Trinité, Silvia Marfil, Cristina Miranda, Sandra González, Ruth Toledo, Marta Font, Susana Benet, Tuixent Escribà, Esther Jimenez-Moyano, Ruth Peña, Samandhy Cedeño, Julia G. Prado, Beatriz Mothe, Christian Brander, Nuria Izquierdo-Useros, Julia Vergara-Alert, Joaquim Segalés, Marta Massanella, Rosa María Benitez, Alba Romero, Daniel Molina-Morant, Julià Blanco, Bonaventura Clotet, Lourdes Mateu, María Luisa Pedro-Botet, Jorge Carrillo

**Affiliations:** 1 IrsiCaixa AIDS Research Institute, Carretera Canyet s/n, Badalona, Catalonia 08916, Spain; 2 Infectious Diseases Department, Germans Trias i Pujol Hospital, Carretera Canyet s/n, Badalona, Catalonia 08916, Spain; 3 Fight AIDS Foundation, Badalona, Catalonia 08916, Spain; 4 Infectious Disease Networking Biomedical Research Center (CIBERINFEC), Carlos III Health Institute, Madrid, Spain; 5 Germans Trias i Pujol Research Institute (IGTP), Carretera Canyet s/n, Badalona, Catalonia 08916, Spain; 6 Catalan Institution for Research and Advanced Studies (ICREA), Barcelona, Catalonia 08010, Spain; 7 University of Vic, Central University of Catalonia, Vic, Catalonia 08500, Spain; 8 Unitat mixta d’Investigació IRTA-UAB en Sanitat Animal, Centre de Recerca en Sanitat Animal (CReSA), Universitat Autonoma de Barcelona, Bellaterra, Catalonia 08193, Spain; 9 IRTA. Programa de Sanitat Animal. Centre de Recerca en Sanitat Animal (CReSA), Universitat Autònoma de Barcelona, Bellaterra, Catalonia 08193, Spain; 10 Departament de Sanitat i Anatomia animal, Facultat de Veterinaria, Universitat Autonoma de Barcelona, Bellaterra, Catalonia 08193, Spain; 11 Respiratory Diseases Networking Biomedical Research Center (CIBERes), Carlos III Health Institute, Madrid, Spain; 12 Universitat Autónoma de Barcelona, Bellaterra, Catalonia 08193, Spain

**Keywords:** Immunology, Virology, Immune response

## Abstract

Mass vaccination campaigns reduced COVID-19 incidence and severity. Here, we evaluated the immune responses developed in SARS-CoV-2-uninfected patients with predominantly antibody-deficiencies (PAD) after three mRNA-1273 vaccine doses. PAD patients were classified based on their immunodeficiency: unclassified primary antibody-deficiency (unPAD, n = 9), common variable immunodeficiency (CVID, n = 12), combined immunodeficiency (CID, n = 1), and thymoma with immunodeficiency (TID, n = 1). unPAD patients and healthy controls (HCs, n = 10) developed similar vaccine-induced humoral responses after two doses. However, CVID patients showed reduced binding and neutralizing titers compared to HCs. Of interest, these PAD groups showed lower levels of Spike-specific IFN-γ-producing cells. CVID individuals also presented diminished CD8^+^T cells. CID and TID patients developed cellular but not humoral responses. Although the third vaccine dose boosted humoral responses in most PAD patients, it had limited effect on expanding cellular immunity. Vaccine-induced immune responses in PAD individuals are heterogeneous, and should be immunomonitored to define a personalized therapeutic strategies.

## Highlights

• PAD individuals mount heterogeneous immune responses to COVID-19 vaccination
• These responses should be monitored to know if additional vaccine doses are needed
• A booster dose increased humoral responses but had limited effect on non-responders
• COVID-19 vaccination induced T-cell responses in most PAD subjects

## Graphical abstract

**Figure:**
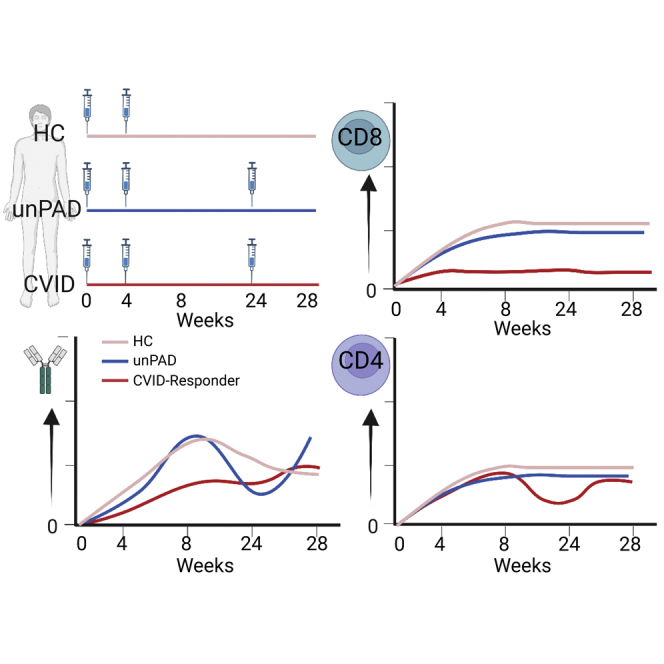

## Introduction

As of May 2022, the severe acute respiratory syndrome coronavirus 2 (SARS-CoV-2) has affected more than 528 million people worldwide, and reached an overall death toll of 6.28 million (https://covid19.who.int/). Fortunately, mass vaccination has drastically reduced the number of SARS-CoV-2-infected individuals that require hospitalization.^1^^,^^2^

Human inborn errors of immunity (IEI) encompass a diverse set of diseases characterized by monogenic germline mutations that result in increased susceptibility to infection, malignant phenotypes, autoimmune, autoinflammatory and allergic diseases,^3^^,^^4^ mainly because of an impaired immune system and specific immunosuppressive treatments (e.g. B cell-depleting agents). IEI patients demonstrate high heterogenicity in their phenotype and clinical manifestation, even in those individuals with identical genetic alterations.^4^ Although these patients were initially considered at risk of severe COVID-19, SARS-CoV-2 seroprevalence and COVID-19-related fatality rate is similar to immunocompetent individuals, and most patients develop mild COVID-19.^5^ However, those IEI individuals that require ICU admission or die because of COVID-19 illness are usually younger than those in the general population.^5^ Severe COVID-19 in IEI individuals has also been associated with comorbidities, including autoimmune or inflammatory complications, lung disease, or higher proinflammatory responses.^5^^,^^6^ Because IEI comprise a highly heterogeneous disease group, severity and fatality rate differ among pathologies.^7^ Particularly, severe combined immunodeficiency, autoimmune polyglandular syndrome type 1, innate immune defect, and Good syndrome are among those IEI groups with higher fatality rate and ICU admission after SARS-CoV-2 infection.^8^

COVID-19 vaccine clinical trials were initially designed to exclude immunocompromised individuals or people receiving immunosuppressive treatment. Thus, there is limited data about COVID-19 vaccine efficacy in IEI patients. Recent studies determined that BNT162b2 vaccine is well tolerated in IEI patients,^9^^,^^10^^,^^11^^,^^12^^,^^13^ and that most patients mount heterogeneous humoral and cellular responses.^9^^,^^10^^,^^11^^,^^12^ For example, whereas individuals with X-linked agammaglobulinemia (XLA) do not generate vaccine-induced antibodies, these patients develop potent cellular responses.^9^^,^^10^^,^^11^ Conversely, SARS-CoV-2-specific immune responses in common variable immunodeficiency (CVID) patients remain controversial. Although two studies reported that the percentage of CVID patients with antigen-specific antibody responses was over 60% after COVID-19 vaccination,^9^^,^^10^ another one only identified 20% responder individuals.^11^ Of interest, CVID patients seroconversion rate is higher after natural infection than vaccination (82 versus 34%, respectively),^13^ and subsequent immunization of convalescent CVID patients can boost their humoral responses. Despite this, some patients remain non-responders.^13^

Although the Center of Disease Control (CDC) started recommending a third vaccine dose to immunocompromised people, its impact on the immune responses of IEI patients has only been partially clarified. Here, we characterized SARS-CoV-2-specific humoral and cellular responses in SARS-CoV-2-uninfected patients with predominantly antibody-deficiencies (PAD) after three mRNA-1273 vaccine doses.

## Results

### Patient characteristics

**Figure 1:**
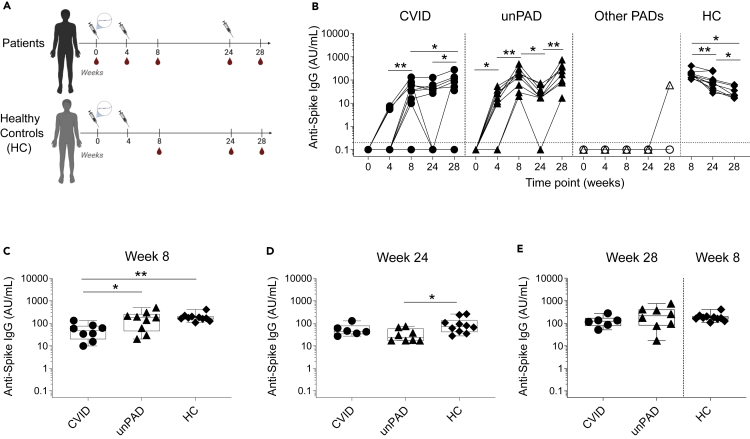
Kinetics of SARS-CoV-2 Spike-specific humoral immune response after vaccination (A) Vaccine regimen timeline and samples collection. (B–D) Anti-Spike IgG levels (in AU/ml) over time. CVID (n = 12, black circles), unPAD (n = 9, black triangles), other PADs (CID: n = 1, open triangles and TID: n = 1, open circles), and HC (n = 10, black diamonds) groups. Data were analyzed using Wilcoxon signed rank test. Vaccine-induced anti-Spike IgG titers in CVID, unPAD and HC responder individuals at w8 (C), and at w24 (D) after the first vaccination. In (B), Horizontal dotted line indicates limit of detection. (E) Vaccine-induced anti-Spike IgG titers in CVID and unPAD responder patients after 28 weeks compared to those elicited at w8 in HCs. In (C–E), Box-whiskers plots showing Min, Max, median and interquartile range are shown. Dunn’s multiple comparison test was utilized for detect differences among groups. ∗p<0.05; ∗∗p<0.01; ∗∗∗p<0.001.

Twenty-seven PAD adult patients under immunoglobulin replacement therapy (IRT) were initially included in the current study. According to 2019 ESID criteria,^14^ patients were classified into four groups: combined immunodeficiency (CID) (1/27), common variable immunodeficiency (CVID) (14/27), thymoma with immunodeficiency (TID) (1/27), and unclassified primary antibody-deficiency (unPAD) (11/27). However, to better define vaccine-induced immune responses in PAD subjects, we excluded four patients (two unPAD, and two CVID individuals) that were diagnosed with SARS-CoV-2 infection either before vaccination or during the length of this study. Of note, anti-nucleocapsid protein (NP) antibodies were detected in one CVID patient after the third vaccine dose. Although this patient did not report signs of SARS-CoV-2 infection, we cannot exclude the possibility of an asymptomatic infection. Therefore, this time point was not considered in our analysis. As expected, none of the remaining 22 SARS-CoV-2-uninfected PAD patients described below showed antibodies against Spike (Figures 1 and S1), receptor-binding domain (RBD) (Figure S2) before immunization, or against NP (data not shown) at any of the analyzed time points, confirming their seronegative status. Healthy controls (HCs, n = 10) also showed lack of reactivity against NP during the length of the current study.

Main characteristics of SARS-CoV-2-uninfected PAD patients are described in Table 1, Table 2 and 2. No severe adverse effects were reported after mRNA-1273 vaccination. A reactogenicity increase was observed after the administration of second vaccine dose, but not after the third immunization (Table S1).

**Table 1:** Patient characteristics

	CVID (n = 12)	UnPAD (n = 9)	CID (n = 1)	TID (n = 1)
Average age (years)	52.25 (29-73)	55.4 (35-71)	72	38
Gender				
Female	3 (25%)	6 (66.6%)	1 (100%)	0 (0%)
Male	9 (75%)	3 (33.3%)	0 (0%)	1 (100%)
Underlying or related diseases				
Lymphoma	2 (16.6%)	0 (0%)	0 (0%)	0 (0%)
Solid cancer				
Basocellular carcinoma	0 (0%)	1 (11.1%)	1 (100%)	1 (100%)
Breast carcinoma	1 (8.3%)	1 (11.1%)	0 (0%)	0 (0%)
Lung adenocarcinoma	0 (0%)	1 (11.1%)	0 (0%)	0 (0%)
Seminoma	0 (0%)	1 (11.1%)	0 (0%)	0 (0%)
Thymoma	0 (0%)	0 (0%)	0 (0%)	1 (100%)
Chronic liver disease	1 (8.3%)	0 (0%)	0 (0%)	0 (0%)
Autoimmune diseases				
Thrombotic thrombocytopenic purpura	2 (16.6%)	0 (0%)	0 (0%)	0 (0%)
Celiac disease	1 (8.3%)	0 (0%)	0 (0%)	0 (0%)
Collagenous colitis	1 (8.3%)	0 (0%)	0 (0%)	0 (0%)
Chron’s disease	1 (8.3%)	0 (0%)	0 (0%)	0 (0%)
Ulcerative proctitis	0 (0%)	1 (11.1%)	0 (0%)	0 (0%)
Autoimmune anemia	1 (8.3%)	0 (0%)	0 (0%)	0 (0%)
Lupus-like syndrome	0 (0%)	1 (11.1%)	0 (0%)	0 (0%)
GLILD	3 (25%)	0 (0%)	0 (0%)	0 (0%)
Asthma	1 (8.3%)	5 (55.5%)	0 (0%)	0 (0%)
Food or drug allergies	3 (25%)	4 (44.4%)	0 (0%)	0 (0%)
Immunosuppressive agents				
Glucocorticoids	1 (8.3%)	0 (0%)	0 (0%)	0 (0%)
Rituximab + glucocorticoids	1 (8.3%)	0 (0%)	0 (0%)	0 (0%)
Rituximab + glucocorticoids + azathioprine	1 (8.3%)	0 (0%)	0 (0%)	0 (0%)
Time since diagnosis (years)	11.8 (4-32)	6.3 (2-16)	3	12
Immunoglobulin deficiency				
IgG	0 (0%)	5 (55.5%)	0 (0%)	0 (0%)
IgG + IgA	2 (16.6%)	2 (22.2%)	0 (0%)	0 (0%)
IgG + IgA + IgM	10 (83.3%)	1 (11.1%)	1 (100%)	1 (100%)
IgG + IgM	0 (0%)	1 (11.1%)	0 (0%)	0 (0%)
IgG subclass deficiency				
1+4	1 (8.3%)	1 (11.1%)	1 (100%)	0 (0%)
1+2+3	1 (8.3%)	0 (0%)	0 (0%)	0 (0%)
1+2+4	6 (50%)	5 (55.5%)	0 (0%)	1 (100%)
1+3+4	0 (0%)	0 (0%)	0 (0%)	0 (0%)
1+2+3+4	2 (16.6%)	3 (33.3%)	0 (0%)	0 (0%)
Not available	2 (16.6%)	0 (0%)	0 (0%)	0 (0%)
Isohemagglutinin levels				
High rates	4 (33.3%)	3 (33.3%)	0 (0%)	0 (0%)
Low rates	7 (58.3%)	3 (33.3%)	0 (0%)	1 (100%)
Not evaluable	1 (8.3%)	0 (0%)	1 (100%)	0 (0%)
Not available	0 (0%)	3 (33.3%)	0 (0%)	0 (0%)
Polysaccharide Typhi Vi antibody response				
Adequate	0 (0%)	5 (55.5%)	0 (0%)	0 (0%)
Not adequate	6 (50%)	3 (33.3%)	1 (100%)	0 (0%)
Not available	6 (50%)	1 (11.1%)	0 (0%)	1 (100%)
Years under IRT	10.8 (3-32)	3.8 (2-5)	2	10
IRT administration route				
Subcutaneous	9 (75%)	2 (22.2%)	1 (100%)	0 (0%)
Intravenous	3 (25%)	7 (77.7%)	0 (0%)	1 (100%)

GLILD, Granulomatous-lymphocytic interstitial lung disease; IRT, immunoglobulin replacement therapy.

**Table 2:** B and T cell levels at the time of immunodeficiency diagnosis

	CVID (n = 12)	UnPAD (n = 9)	CID (n = 1)	TID (n = 1)
Average IgG levels prior to vaccination	813.75 (638-1013)	815.6 (598-1112)	622	800
Absolute B cell count (cells/μL)	103.58 (0-214)	169.4 (74-329)	76	25
B cell frequency	7.1% (0-13.8)	8.6% (5.5-13.7)	12.9%	23.2%
Memory B cell frequency	13.8% (0-30.6)	17.9% (2.6-28.3)	5.7%	2.3%
IgM+ memory B cell frequency	2.1% (0-10)	3.5% (0.1-15.5)	1.9%	0%
Transitional B cell frequency	6.7% (0.1-39.1)	6% (0.2-15.1)	18.3%	32.4%
Switched B cell frequency	2.1% (0-18.9)	18.2% (0.5-59.2)	8.6%	0%
Absolute CD8^+^ T cell count (cells/μL)	557.9 (169-1360)	611.2 (354-1598)	56	1068
CD8^+^ T cell frequency	35.9% (16.9-54.4)	28.4% (16.2-37.7)	11.3%	56.2%
Absolute CD4^+^ T cell count (cells/μL)	593.9 (252-1476)	960.1 (396-1598)	105	500
CD4^+^ T cell frequency	36.6% (22.9-58.9)	47.1% (33.4-64.1)	21%	26.3%

### COVID-19 vaccination induces heterogeneous SARS-CoV-2-specific humoral response in PAD patients

To determine how PAD individuals respond to COVID-19 mRNA vaccine, we analyzed the humoral responses elicited against Spike (Figure 1) and RBD (Figure S2) in 23 SARS-CoV-2-uninfected PAD vaccinees (Figure 1A). Of note, HCs did not receive the third vaccine dose at w24 (Figure 1A). Although all HCs developed Spike-specific IgG at week 8 (w8) (Figure 1B), IgG seroconversion was observed in 25% (3/12) and 67% (8/12) of CVID patients at w4 and w8, respectively (Figure 1B). Average IgG levels in CVID responders at w8 (53 ± 40 AU/mL) were still significantly lower than those observed in HCs (191 ± 83 AU/mL, p = 0.007, Figure 1C). Of the CVID patients that showed antigen-specific IgG at w8, 25% (2/8) became undetectable after six months, whereas 75% (6/8) were able to sustain their titers (Figures 1B and 1D). Most CVID individuals that had a detectable antigen-specific IgG response at w8 (67%) showed a rise in antibody levels four weeks after the third immunization (w8: 53 ± 40 AU/mL; w28: 114 ± 87 AU/mL, p = 0.03, Figure 1B). Despite this, 42% (5/11) of CVID individuals remained IgG seronegative at w28. Because one CVID patient showed anti-NP IgG antibodies, and might have become infected at w28, we excluded this value from the analysis.

Conversely, 78% (7/9) and 100% (9/9) of unPAD individuals seroconverted at w4 and w8, respectively Figure 1B). Anti-Spike IgG levels in unPAD responders at w8 (181 ± 149 AU/mL) were similar to those observed in HCs (191 ± 83 AU/mL, p>0.99), and significantly higher than in CVID responders (p = 0.04, Figure 1C). A significant decrease in antibody levels was observed from w8 to w24 in both HC (191 ± 83 AU/mL versus 100 ± 81 AU/mL, p = 0.006) and unPAD groups (181 ± 149 AU/mL versus 34 ± 23 AU/mL, p = 0.004, Figure 1B). Remarkably, and even though unPAD antibody levels were similar at w8 to the HC group (Figure 1C), these individuals showed lower IgG levels at w24 (p = 0.02, Figure 1D). Despite that, only 11% (1/9) of unPAD patients were below the detection limit at w24. Administration of the third vaccine dose boosted IgG levels in all unPAD patients (p = 0.008) to similar levels than those observed at w8 in the HCs (p>0.99, Figure 1E). We were unable to detect antigen-specific IgG in patients with CID or TID at w8 or w24 (Figures 1B and S2). However, the CID patient seroconverted after the third dose (60 AU/mL), showing the potential benefit of this additional shot.

To characterize the humoral responses developed after vaccination in our cohort of PAD patients, we also assessed the presence of anti-Spike IgA and IgM in circulation (Figure S1). At w8, we identified anti-Spike IgA in 90% (9/10) of HCs, whose levels remained stable over time, 8% (1/12) of CVID, and 67% (6/9) of unPAD patients. Although IgA levels were sustained in HCs, they decreased in PAD responders from w8 to w24 (w8: 12 ± 19, w24: 4 ± 6, p = 0.016). Of interest, the third vaccine dose boosted IgA in these individuals (w28: 27 ± 45; p = 0.031, Figure S1A). Nonetheless, the majority of CVID patients (92%) remained IgA negative during the length of this study. IgM responses were only observed in 33% (3/10) of HCs, 17% (2/12) of CVID, and 44% (4/9) of unPAD individuals (Figure S1A). Only 50% of IgM PAD responders were able to maintain these responses over time. The third vaccine dose had little effect on IgM titers (Figure S1A). It is noteworthy to mention that of the five CVID patients that had undetectable Spike-specific IgG at w28, one of them was IgM positive at w8 and w28, and another one elicited low levels of IgM at w28. We were unable to detect Spike-specific IgA in any of these patients throughout the course of this study.

**Figure 2:**
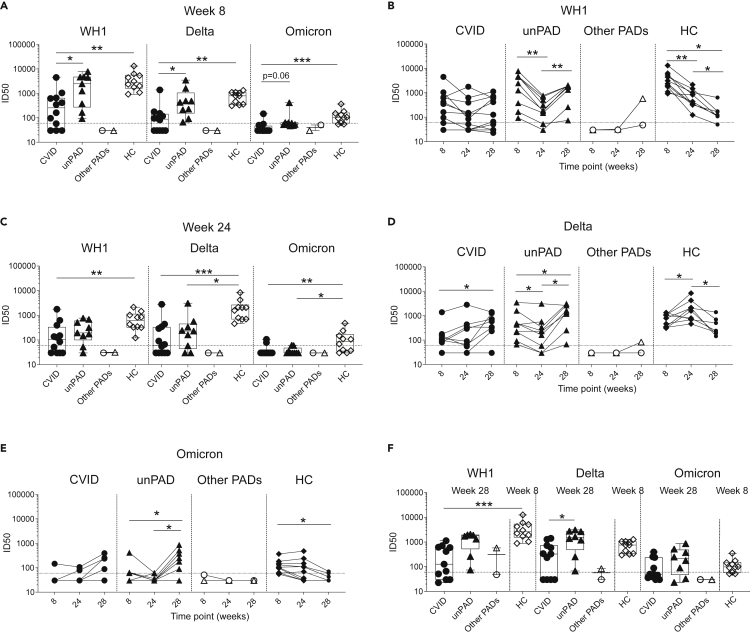
Vaccine-induced neutralizing activity against WH1, Delta, and Omicron in PAD patients (A) NAb ID50 titers elicited in CVID (n = 12, black circles), unPAD (n = 9, black triangles), other PADs (CID: n = 1, open triangles and TID: n = 1, open circles), and HC groups (n = 10, black diamonds) against SARS-CoV-2 WH1, Delta, and Omicron variants at w8. (B) Time course of vaccine-induced neutralizing antibodies in all groups against WH1 variant. (C–E) Levels of NAbs at w24 in PAD patients and HC group against WH1, Delta, and Omicron variants, respectively. NAb titers against Delta (D) and Omicron (E) elicited in CVID, unPAD, other PADs, and HCs. (F) NAb levels after three vaccine doses (w28) in PAD patients compared to those elicited after two doses (w8) in HCs. ID50: Half maximal inhibitory dilution. In (A,C, and F), Box-whiskers plots showing Min, Max, median and interquartile range are shown. Data was analyzed using Dunn’s Multiple Comparison Test (A, C and F), and Wilcoxon signed rank test (B, D and E). ∗p<0.05; ∗∗p<0.01; ∗∗∗p<0.001; ∗∗∗∗p < 0.0001. Horizontal dotted line indicates limit of detection.

Anti-Spike IgG responses correlated with RBD-specific IgG levels (Figure S2D). Because RBD is considered a major target of neutralizing antibodies (NAbs),^15^^,^^16^ we evaluated the capacity of our 23 SARS-CoV-2-uninfected PAD vaccinees to neutralize the ancestral SARS-CoV-2 Wuhan-Hu-1 (WH1), and two additional variants of concern (VoC): Delta (B.1.617.2) and Omicron (B.1.1.529). We detected higher titers of NAbs against both WH1 and Delta VoC in HCs (WH1: 4047 ± 3657; Delta: 762 ± 376), and unPAD patients (WH1: 2892 ± 2680; Delta: 786 ± 1107) than in CVID individuals at w8 (WH1: 664 ± 1247, Delta: 188 ± 382) (Figure 2A). Low NAb titers against Omicron were measured in HCs at w8 (131 ± 93, Figure 2A). However, this neutralizing activity was hardly detected in CVID and unPAD individuals. According to our binding data (Figure 1C), the NAb titers against WH1 waned over time in HC (p = 0.002) and unPAD groups (p = 0.004), even though they remained stable in CVID patients (p = 0.1, Figure 2B). Despite that, HCs showed higher levels of neutralization at w24 against WH1 than CVID group (p = 0.001) and against Delta and Omicron than unPAD (p = 0.019, p = 0.035, respectively) and CVID groups (p = 0.001, p = 0.006, respectively) (Figure 2C). Of interest, whereas NAb titers decreased in unPAD individuals, and were sustained in CVID patients over time, a transient increase was observed in HCs at w24. After that, NAbs decreased in the absence of an additional vaccine dose (Figure 2D). Anti-Omicron neutralization titers also waned over time, but were still detected in 50% (5/10) of HCs at w24 (Figures 2C and 2E), becoming practically undetectable at w28.

The third vaccine dose increased neutralization levels against all variants in the unPAD group (WH1: p = 0.008, Delta: p = 0.04, Omicron: p = 0.03, Figures 2B, 2D, and 2E), who recovered their WH1-specific NAb titers observed at w8. After boosting, unPAD patients showed higher NAb titers against all VoC than those elicited at w8 (Delta: 786 ± 1107 versus 1626 ± 1138, p = 0.03; Omicron: 96 ± 120 versus 291 ± 288, p = 0.03, Figures 2D and 2E), and similar neutralizing activity to the ones observed in HCs (Figure 2F). No impact on the NAb titers was observed in CVID (p>0.1, Figures 2B and 2F). In line with previous binding data (Figure 1B), poor neutralizing activity was observed in the CID patient at w28, probably because of the presence of low IgG levels.

**Figure 3:**
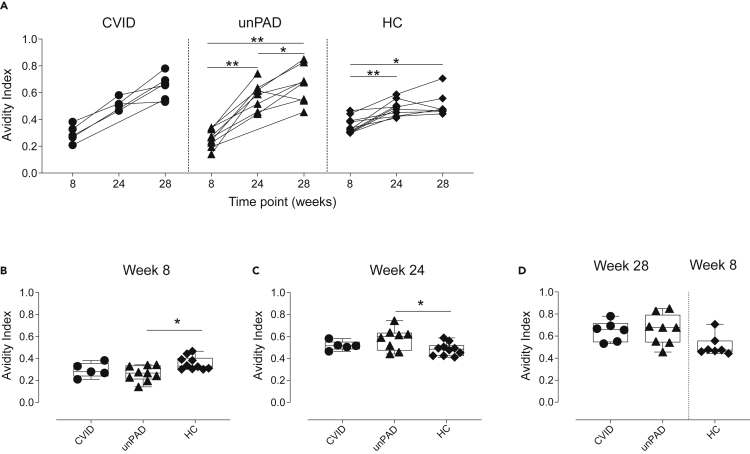
Avidity of vaccine-induced Spike-specific IgG binding (A–C) Anti-Spike IgG avidity over time in vaccinated CVID (black circles, n = 6), unPAD (black triangles, n = 9), and HCs (black diamonds, n = 10). Comparison of anti-Spike IgG avidity in vaccinated CVID, unPAD, and HCs at w8 (B), and w24 (C). (D) Comparison of anti-Spike IgG avidity in vaccinated CVID, unPAD at w28 and HCs at w8. In (B–D), Box-whiskers plots showing Min, Max, median and interquartile range are shown. Data in (A) was analyzed using Wilcoxon signed rank test. Data in (B–D) were analyzed using Dunn’s Multiple Comparison Test. ∗p<0.05; ∗∗p<0.01.

We then evaluated anti-Spike IgG avidity in a subset of PAD patients who responded to vaccination, and observed that IgG avidity significantly increased in both unPAD and HC groups over time (Figure 3A). A similar positive trend was observed in CVID patients. Of interest, although unPAD patients had similar levels of anti-Spike IgG to HCs (Figure 1C), they showed reduced IgG avidity at w8 (0.26 ± 0.07 versus 0.36 ± 0.06, p = 0.044, Figure 3B). Conversely, these patients developed higher IgG avidity than HCs at w24 (0.58 ± 0.1 versus 0.48 ± 0.06, p = 0.049, Figure 3C). After the third dose, antigen-specific IgG avidity in CVID and unPAD patients continued to increase, reaching similar values (0.65 ± 0.09 versus 0.66 ± 0.14, p>0.99, Figures 3A and 3D).

### Induction of low levels of SARS-CoV-2-specific cellular responses in PAD patients after COVID-19 vaccination

**Figure 4:**
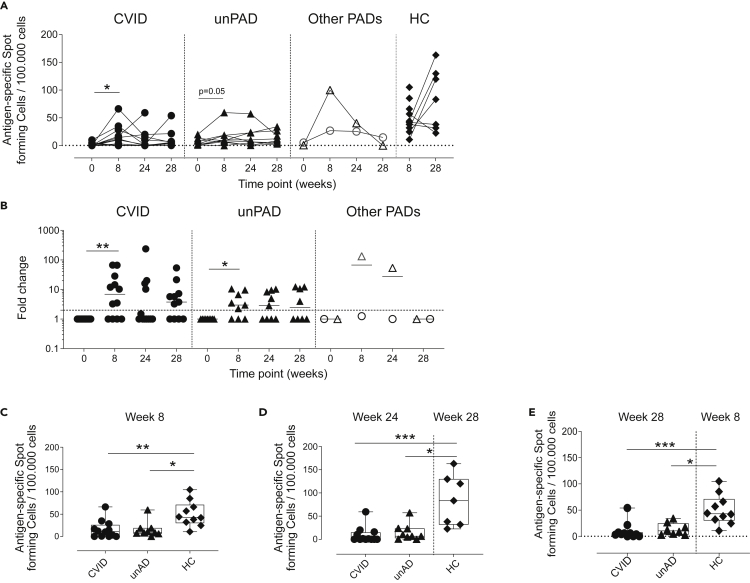
Vaccine-induced SARS-CoV-2-specific IFN-ɣ T cell responses (A) Number of antigen-specific IFN-ɣ-producing T cells in CVID (n = 12, black circles), unPAD (n = 9, black triangles), other PADs (CID: n = 1, open triangles and TID: n = 1, open circles), and HC groups (n = 10, black diamonds) per 100.000 cells. (B–E) Antigen-specific IFN-ɣ-producing T cells fold change respect to basal (w0). Comparison of Spike-specific spot-forming cells among CVID, unPAD, and HC groups at w8 (C), w24 versus w28 (D), and w28 versus w8 (E). In (C–E), Box-whiskers plots showing Min, Max, median and interquartile range are shown. (A and B) Horizontal dotted line indicates limit of detection and in (B) lines indicate median. Data in (A and B) were analyzed using Wilcoxon signed rank test. Data in (C–E) were analyzed using Dunn’s Multiple Comparison Test. ∗p<0.05; ∗∗p<0.01; ∗∗∗p < 0.001.

Next, we evaluated vaccine-induced cellular responses against Spike by IFN-γ ELISpot and flow cytometry at w0, w8, w24, and w28 (Figure S3A). All HCs showed high levels of Spike-specific IFN-γ-producing cells at w8 (50 ± 28 SFC/10^5^ cells) and w28 (84 ± 55 SFC/10^5^ cells), indicating that these responses were stable and, in some cases, increased over time (Figure 4A). Conversely, IFN-γ responses were detected in 67% (8/12) of CVID patients at w8 (23 ± 20 SFC/10^5^ cells, p = 0.01, Figures 4A and 4B). Six months later, we observed IFN-γ responses in only 33% (4/12) of CVID individuals. Of interest, the administration of the third vaccine dose restored the frequency of CVID patients that showed IFN-γ-producing cells to those levels observed at w8 (15 ± 19 SFC/10^5^ cells, Figures 4A and 4B). Similarly, 67% (6/9) of unPAD patients developed IFN-γ-producing cells after two doses (21 ± 20 SFC/10^5^ cells, p = 0.05, Figures 4A and 4B), and five of them remained detectable at w24 (24 ± 20 SFC/10^5^ cells). The third vaccine dose had no effect in this group (p>0.1, Figures 4A and 4B). Of note, the magnitude of these responses in CVID and unPAD groups was lower than HCs at w8 (p = 0.007, p = 0.014, respectively) and w24, compared with HCs at w28 (p = 0.001, p = 0.044, respectively). After the third vaccine boost (w28), these responses were still lower than those observed in HCs at w8 (p = 0.001, p = 0.025, respectively, Figures 4C–4E). Although both CID and TID patients did not develop antigen-specific IgG after two doses, these individuals showed detectable IFN-γ-producing cells at w8 (Figure 4A). Particularly, the CID patient showed a large IFN-γ-producing response at w8, which progressively declined until w24. No boost was observed in these patients after the third vaccine dose (Figures 4A and4B).

**Figure 5:**
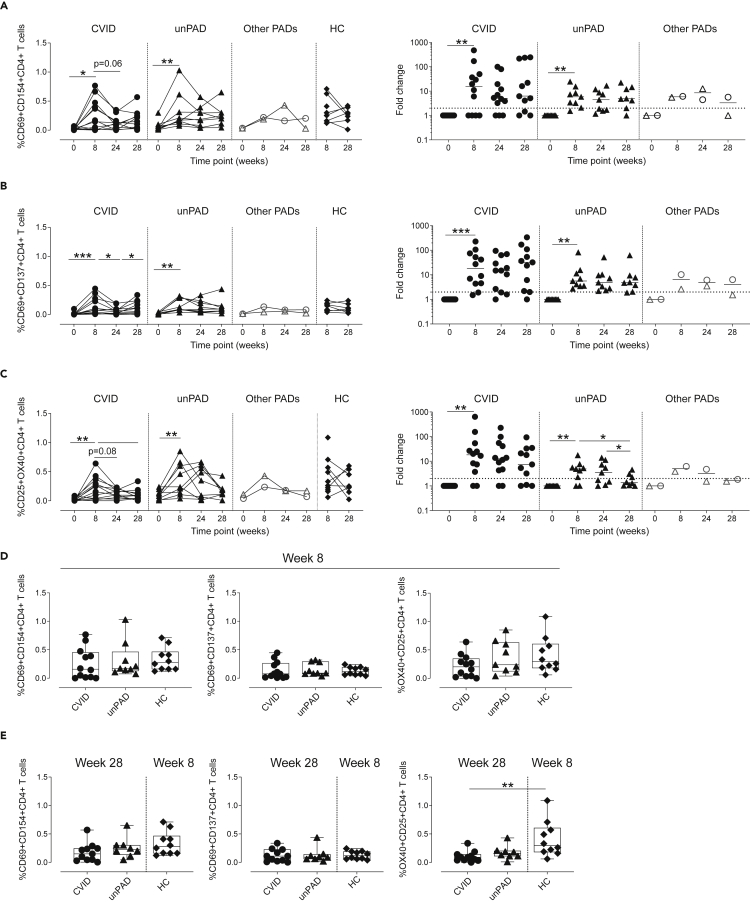
Frequency of Spike-specific CD4^+^T cell using activation-induced markers (A–E) Frequency (left panel) or fold change (right panel) in respect to w0 of Spike-specific CD4+T cells expressing CD69+CD154+ (A), CD69+CD137+ (B) or CD25+OX40+ (C) in CVID (n = 12, black circles), unPAD (n = 9, black triangles), other PADs (CID: n = 1, open triangles and TID: n = 1, open circles), and HC groups (n = 10, black diamonds) after vaccination. Comparison of Spike-specific CD4^+^T cell subsets in CVID, unPAD and HC groups at w8 (D), and w28 versus w8 (E). In (D and E), Box-whiskers plots showing Min, Max, median and interquartile range are shown. (A–C) Horizontal dotted line indicates limit of detection and lines indicate median. Data in (A–C) were analyzed using Wilcoxon signed rank test. Data in (D and E) were analyzed using Dunn’s Multiple Comparison Test. ∗p<0.05; ∗∗p<0.01; ∗∗∗p < 0.001.

**Figure 6:**
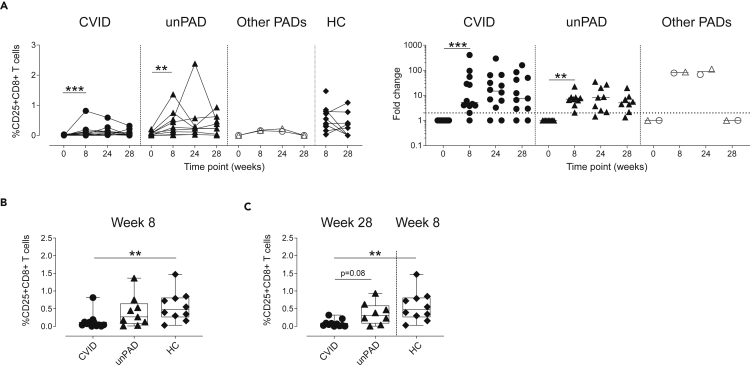
Vaccine-induced SARS-CoV-2-specific CD8+T cells (A–C) Frequency of Spike-specific CD25+CD8+T cell (left panel) and fold change (right panel) in CVID (n = 12, black circles), unPAD (n = 9, black triangles), other PADs (CID: n = 1, open triangles and TID: n = 1, open circles), and HC groups (n = 10, black diamonds) after vaccination. Comparison of CD25+CD8+T cell frequency among CVID, unPAD, and HC groups at w8 (B) and w28 versus w8 (C). In (B), Box-whiskers plots showing Min, Max, median and interquartile range are shown and in (A) lines indicate median. Data in (A) was analyzed using Wilcoxon signed rank test. Data in (B and C) were analyzed using Dunn’s Multiple Comparison Test. ∗p<0.05; ∗∗p<0.01; ∗∗∗p < 0.001. Horizontal dotted line indicates limit of detection.

We then analyzed activation-induced markers by flow cytometry in both CD4+ and CD8+T cells after stimulation with S1 peptides (Figure S3). We observed a significant increase at w8 in the frequency of S1-specific CD69+CD154+CD4+ (Figure 5A), CD69+CD137+CD4+ (Figure 5B) and CD25+OX40+CD4+T cells in CVID (p = 0.03, p = 0.0005, p = 0.005, respectively) and unPAD patients (p = 0.004, p = 0.004, p = 0.008, respectively, Figure 5C). The magnitude of the CD4+ subsets analyzed at w8 in CVID and unPAD groups was similar to those observed in HCs (p>0.3, Figure 5D), and remained stable in the unPAD group until w24 (Figures 5A–5C). Similar results were observed in CID and TID patients. However, when the CVID group was analyzed, we observed a reduction in the frequency of CD69+CD137+CD4+T cells (p = 0.02), and a decreasing trend in both CD69+CD154+CD4+ (p = 0.06) and CD25+OX40+CD4+T cells (p = 0.08) from w8 to w24 (Figures 5A–5C). Remarkably, the third vaccine dose did not significantly boost CD4+T cell responses in unPAD patients (p>0.99, Figure 5E). However, a frequency increase of CD69+CD137+CD4+T cells was observed in CVID patients after the third vaccine dose (p = 0.04, Figure 5B), reaching similar values than those detected in HCs at w8 (p>0.99, Figure 5E). Despite that, lower frequency of CD25+OX40+CD4+T cells in CVID patients at w28 was observed when compared to HCs (p = 0.003, Figure 5E). Of interest, 92% of CVID (11/12, p = 0.001) and 100% of unPAD patients (9/9, p = 0.004) elicited CD25+CD8+T cells at w8, which remained stable in all groups (Figure 6A). Despite that, the magnitude of CD25+CD8+T cells in CVID patients was significantly lower than in HCs at w8 (p = 0.003, Figure 6B). The administration of the third vaccine dose did not boost CD8+T cells responses in CVID or unPAD individuals (Figure 6A), which remained significantly lower than in HCs at w8 (p = 0.002, Figure 6C). Similar results were observed in CID and TID patients (Figure 6A).

## Discussion

Although the three-dose COVID-19 mRNA vaccine regimen has shown increased effectiveness over the two original doses in healthy individuals,^17^ its effect remains largely unknown in PAD individuals. Here, we characterized the immune responses elicited in 23 SARS-CoV-2-uninfected PAD patients after receiving three mRNA-1273 vaccine doses. Although our PAD cohort is mainly composed of unPAD and CVID patients, it also includes one CID and TID patient, which might be interesting because of the limited available information about how these individuals respond to COVID-19 vaccination.^18^^,^^19^ According to previous reports,^10^^,^^11^^,^^12^^,^^18^^,^^19^^,^^20^^,^^21^ our results showed that mRNA-1273 immunization was safe, and most patients developed Spike-specific immune responses. However, the heterogenicity of PAD disorders is reflected in the distinct immune responses elicited after vaccination. For example, although unPAD patients developed vaccine-induced humoral responses with similar kinetics to those elicited in HCs, they showed a faster decline of NAb titers over time, requiring a third vaccine dose to develop NAbs against Omicron, which was achieved in HCs after two doses. Thus, COVID-19 vaccination efficacy may wane earlier in unPAD patients than in the general population. In contrast, only 67% of CVID individuals seroconverted at w8, and developed lower levels of anti-Spike IgG and NAb titers against all VoC than HC and unPAD groups. Of interest, antibody levels remained stable over time in most CVID responders. Although vaccine-induced IgA responses were detected in most unPAD and HC individuals, only one CVID patient elicited anti-Spike IgA. These results were not surprising, because most CVID individuals, including the ones in our cohort, demonstrate impaired IgA responses.^22^ Notably, we detected low levels of anti-Spike IgM in two CVID patients who had not developed IgG or IgA responses. One of them showed low NAb titers against WH1 and Delta. Differences observed in the humoral responses among groups may be because of the enrollment of different B cell subsets. Particularly, the generation of antibody-secreting cells (ASCs) with distinctive half-life (short to intermediate versus long) could explain why antibody levels waned differently in PAD and HC groups. It has been previously described that the frequency of T and B cell subsets in unPAD individuals is comparable to HCs,^23^ which could explain the similar kinetics observed between these two groups. Remarkably, although humoral responses in the CVID group were more heterogeneous, the stability of the antibody levels observed in responder individuals suggests that long-lived ASCs might be generated in a fraction of them. These results are noteworthy because it has been described that CVID individuals demonstrate a dysregulated B cell compartment, characterized by a reduction of class-switched memory B cells, and an increased frequency of atypical memory B cells (CD19+CD27-CD21-IgM-IgD-), which encompasses most Spike-specific memory B cells after COVID-19 vaccination.^11^ Although the origin of these cells remains unclear, it has been postulated that they might derive from an extrafollicular B cell response, a T-independent response, or an early germinal center (GC) reaction.^11^^,^^24^ Because the avidity of anti-Spike IgG responses gradually increased after the second vaccine dose in CVID responders, our results support a potential involvement of GCs and antigen-specific B cell selection in a subset of CVID patients, that could contribute to the generation of long-lived ASCs. In line with that, COVID-19 vaccination induces a persistent GC reaction in healthy individuals,^25^ and it is indeed possible to detect GCs and somatic hypermutations in CVID patients.^26^^,^^27^ However, GCs in CVID individuals might be dysfunctional, which could explain the increased frequency of atypical memory B cells.^27^

In addition to characterize the humoral responses in our PAD cohort, we defined the vaccine-induced cellular responses using two assays: IFN-γ ELISpot, and the detection of activation markers by flow cytometry.^28^ Both assays showed that cellular responses were sustained in unPAD and HC groups, and that the third vaccine dose had no effect on expanding the magnitude of previously-generated responses in unPAD individuals. Contrarily, although CD4+T cell responses decreased in CVID patients over time, these responses were boosted after the third immunization. Compared to HCs, CVID patients developed lower frequency of CD8+T cell responses that remained stable over time. Intriguingly, we observed a discrepancy in the magnitude of antigen-specific cellular responses detected by both techniques. We identified significantly lower levels of IFN-γ-secreting T cells in CVID and unPAD patients compared to HCs after two and three vaccine doses. These differences were not detected by flow cytometry, except in OX40+CD25+CD4+T cells of CVID patients at w28, which were lower than HCs. Our results may suggest that although T cell responses could be generated after vaccination in unPAD and CVID individuals, their function (i.e. IFN-γ production) might be impaired. Accordingly, Fernandez et al. described a lower proportion of IFN-γ responses after stimulation with Spike-derived peptides in COVID-19 vaccinated CVID patients,^11^ which could be a general particularity of CVID individuals.^29^ Conversely, Hagin et al.^10^ stated that vaccine-induced cellular responses in IEI patients and HCs were similar. Although IEI patients in this latter study and ours are different, ELISpot data obtained in the CVID group from Hagin et al., and the one presented herein seem comparable. It is possible that the discrepancy lies in that our HCs showed greater levels of IFN-γ-producing responses than those described in Hagin et al.^10^

Besides unPAD and CVID patients, we also analyzed the vaccine-induced immune responses in one CID and one TID patients. Although none of them showed anti-Spike antibodies, both patients developed Spike-specific cellular responses after two vaccine doses. Similarly, we identified detectable T cell responses in three of five CVID patients who did not elicit humoral responses. It has been previously described that individuals who do not develop humoral responses after vaccination (e.g. XLA individuals^11^ or patients treated with anti-CD20 antibodies^30^) can elicit antigen-specific cellular responses. Of interest, although the CID patient seroconverted after receiving a third dose, showing Spike-specific IgG and anti-WH1 NAbs, we were unable to detect Spike-specific immune responses in two CVID patients after three COVID-19 immunizations. Immunosuppressive therapies have been associated with the development of poor humoral responses in patients with multiple sclerosis,^31^ limiting the efficacy of COVID-19 mRNA vaccines.^32^^,^^33^ However, of the two CVID subjects who did not elicit vaccine-induced immune responses, only one received immunosuppressive treatment. Thus, because of the small sample size, we cannot conclude the impact of these therapies in halting vaccine-induced immunity in our study.

Our study highlights the distinct response to COVID-19 vaccination elicited in PAD individuals. Although most individuals mount Spike-specific immune responses, there is a fraction of subjects that remained non-responders even after three vaccine doses. Therefore, immunomonitoring of these patients could provide insights about their immune status and the need of additional vaccine doses or other prophylactic approaches.

### Limitations of the study

Although our results regarding immune responses in PAD patients are similar to other published work,^10^^,^^11^^,^^12^^,^^18^^,^^20^^,^^21^ the heterogenicity of our cohort and its small size, in addition to other several factors (i.e. patient heterogenicity, methodology, time points, and administered vaccine), hinder direct comparison among studies.

## STAR★Methods

### Key resources table

**Table:** 

REAGENT or RESOURCE	SOURCE	IDENTIFIER
**Antibodies**		

6x-His Tag Monoclonal Antibody (HIS.H8)	ThermoFisher Scientific	Cat# MA1-21315; RRID AB_557403
Peroxidase AffiniPure F(ab')₂Fragment Goat Anti-Human IgG, Fcγ fragment specific	Jackson ImmunoResearch	Cat# 109-036-098; RRID: AB_2337596
Peroxidase AffiniPure Goat Anti-Human Serum IgA, α chain specific	Jackson ImmunoResearch	Cat# 109-035-011; RRID: AB_2337580
Peroxidase AffiniPure F(ab')₂ Fragment Goat Anti-Human IgM, Fc5μ fragment specific	Jackson ImmunoResearch	Cat# 109-036-129; RRID: AB_2337598
CD40 Antibody, anti-human, pure-functional grade clone HB14	Miltenyi Biotec	Cat# 130-094-133; RRID:AB_10839704
CD49d (Integrin alpha 4) Monoclonal Antibody Clone 9F10, Functional Grade	ThermoFisher Scientific	Cat# 16-0499-85; RRID: AB_468973
BD Horizon™ R718 Mouse Anti-Human CD5 clone UCHCT2	BD Biosciences	Cat# 567058; RRID:AB_2916409
BD Pharmingen™ APC-H7 Mouse anti-Human CD8 clone SK1	BD Biosciences	Cat# 560179; RRID: AB_1645481
BD Horizon™ BV605 Mouse Anti-Human CD4 clone RPA-T4	BD Biosciences	Cat# 562658; RRID: AB_2744420
BD Horizon™ BV421 Mouse Anti-Human CD25 clone 2A3	BD Biosciences	Cat# 564033; RRID: AB_2738555
BD Horizon™ V500 Mouse Anti-Human CD14 clone M5E2	BD Biosciences	Cat# 561391; RRID: AB_10611856
BD Horizon™ V500 Mouse anti-Human CD19 clone hib19	BD Biosciences	Cat# 561121; RRID: AB_10562391
BD OptiBuild™ BB700 Mouse Anti-Human CD154 (CD40L) clone trap-1	BD Biosciences	Cat# 745814; RRID: AB_2743265
BD Pharmingen™ APC Mouse Anti-Human CD137 clone 4B4-1	BD Biosciences	Cat# 550890; RRID: AB_398477
PE/Cyaine7 anti-human CD134 (OX40) Antibody clone Ber-ACT35 (ACT35)	BioLegend	Cat# 350012; RRID: AB_10901161
PE anti-human CD69 Antibody clone FN50	BioLegend	Cat# 310906; RRID: AB_314841

**Bacterial and virus strains**		

pNL4-3.Luc.R-.E−	NIH ARP	Cat# 3418
SARS-CoV-2.SctΔ19	Paper	Pradenas et al.,^34^ Med NY 2021
pcDNA3.4-TOPO	GeneArt/Thermo Fisher Scientific	Cat# 810330DE
pVSV-G	Clontech	Sánchez-Palomino et al., Vaccine 2011

**Biological samples**		

Participant sera	This study	N/A
ELISA standard, positive plasma sample	This study	N/A

**Chemicals, peptides, and recombinant proteins**		

SARS-CoV-2 (2019-nCoV) Spike S1+S2 ECD-His Recombinant Protein	Sino Biological Inc	Cat# 40589-V08B1
SARS-CoV-2 (2019-nCoV) Spike RBD-His Recombinant Protein	Sino Biological Inc	Cat# 40592-V08H
SARS-CoV-2 (2019-nCoV) Nucleocapsid-His recombinant Protein	Sino Biological Inc	Cat# 40588-V08B
Guanidine hydrochloride solution BioUltra, ∼8 M in H2O	Merck Life Science SLU	Cat# 50937
MACS BSA solution	Miltenyi Biotec	Cat# 130-091-376
Phosphate Buffered Saline	Thermo Fisher Scientific	Cat# 10010015
o-Phenylenediamine dihydrochloride	Merck Life Science SLU	Cat# P8787-100TAB
H_2_SO_4_	Sigma-Aldrich	Cat# 258105-1L-PC-M
Fetal Bovine Serum	Thermo Fisher Scientific	Cat# 10270106
Dulbecco’s Modified Eagle Medium	Thermo Fisher Scientific	Cat# 41966052
Expi293 Expression Medium	Thermo Fisher Scientific	Cat# A1435102
Opti-MEM I Reduced Serum Medium	Thermo Fisher Scientific	Cat# 31985070
ExpiFectamine 293 Transfection Kit	Thermo Fisher Scientific	Cat# A14524
Versene	Thermo Fisher Scientific	Cat# 15040033
Puromycin	Thermo Fisher Scientific	Cat# A1113803
DEAE-Dextran	Sigma-Aldrich	Cat# D9885-100G
BriteLite Plus Luciferase	PerkinElmer	Cat# 6066769
CytoStim™, human	Miltenyi Biotec	Cat# 130-092-173
PepTivator ® SARS-CoV-2 Prot_S1, research grade, RG 60nmol	Miltenyi Biotec	Cat# 130-127-048
Human TruStain FcX™ (Fc Receptor Blocking Solution)	BioLegend	Cat# 422301

**Critical commercial assays**		

ELISpot Flex: Human IFN-γ (ALP)	Mabtech	Cat# 3420-2A
AP Conjugate Substrate Kit	BioRad	Cat# 1706432
LIVE/Dead™ Fixable Aqua - Dead Cell Stain Kit (405 nm)	ThermoFisher Scientific	Cat# L34957

**Experimental models: Cell lines**		

Expi293F GnTI- cells	Thermo Fisher Scientific	Cat# A39240
HEK293T/hACE2 cells	Integral Molecular	Cat# C-HA101

**Software and algorithms**		

FlowJo (Treestar)	BD Biosciences	https://www.flowjo.com
GraphPad Prism v8.0	GraphPad Software	https://www.graphpad.com/scientific-software/prism/

### Resource availability

#### Lead contact

Further information and requests for resources and reagents should be directed to and will be fulfilled by the lead contact, Jorge Carrillo (jcarrillo@irsicaixa.es).

#### Materials availability

This study did not generate new unique reagents.

### Experimental model and subject details

#### Study overview and human subjects

A prospective observational cohort-comparative study was conducted at the Hospital Universitari Germans Trias i Pujol (Badalona, Spain) with previous Institutional Review Board approval (PI-21-107). We included 27 PAD patients (age >18 years) that had received IRT. Patients with the following conditions were excluded: previous SARS-CoV-2 vaccination, vaccine-induced anaphylactic reaction, allergy to polysorbate or polyethylene glycol, as well as pregnant or breastfeeding women. Samples from 10 SARS-CoV-2-uninfected COVID-19-vaccinated HCs (age and gender balanced) were included for comparative purposes. All participants provided written informed consent. Patients and HCs were administered with two doses of mRNA-1273 (Moderna) vaccine. A third dose was administered to all PAD patients at w24 after first immunization.

#### Cell lines

HEK293T cells (presumably of female origin) overexpressing WT human ACE-2 (Integral Molecular, USA). Cells were cultured in T75 flasks with Dulbecco′s Modified Eagle′s Medium (DMEM) supplemented with 10% FBS and 1 μg/mL of puromycin (Thermo Fisher Scientific, USA). HEK293T/hACE2 cells were used as target for SARS-CoV-2 Spike-expressing pseudovirus infection.

### Method details

#### Samples collection

Blood samples were collected at w0, w4, w8, w24, and/or w28 post-first dose in EDTA tubes. Peripheral blood mononucleated cells (PBMCs) were isolated by standard density-gradient centrifugation using Ficoll-Paque (Atom Reactiva) and cryopreserved in liquid nitrogen. Plasma was obtained after blood centrifugation and stored at −80°C until use.

#### SARS-CoV-2-specific IgG, IgA, and IgM ELISA

Anti-SARS-CoV-2 IgG, IgA, and IgM antibodies were quantified using an in-house developed sandwich-ELISA. Nunc MaxiSorp ELISA plates were coated overnight at 4°C with an anti-6xHis antibody (clone HIS.H8; Thermo Fisher Scientific) at 2 μg/mL, and blocked with PBS/1% BSA (Miltenyi Biotec). The following SARS-CoV-2 antigens were added to half of the plate at a concentration of 1 μg/mL: Spike, RBD or NP (Sino Biological). The other half of the plate received PBS/1% BSA. Heat-inactivated plasma samples (56°C for 40 min) were assessed in quadruplicate. Two wells contained SARS-CoV-2 antigens, and the other two received PBS/1% BSA. A serially diluted positive plasma sample was used as standard, and a pool of pre-pandemic SARS-CoV-2-uninfected samples were utilized as negative control. The different isotypes were detected using the following secondary antibodies: HRP-conjugated goat anti-human IgG (1/20,000), goat anti-human IgM (1/10,000), and goat anti-human IgA (1/10,000) (all from Jackson ImmunoResearch), which were incubated for 30 min at room temperature (RT). After washing, plates were exposed using o-phenylenediamine dihydrochloride (OPD) (Sigma Aldrich), and the enzymatic reaction was stopped with 2M of H_2_SO_4_ (Sigma Aldrich). The signal was analyzed as optical density (OD) at 492 nm with noise correction at 620 nm. Antigen-specific signal was calculated by subtracting background obtained for each sample in antigen-free wells. Results are shown as arbitrary units (AU)/mL.

#### IgG avidity ELISA

Nunc MaxiSorp ELISA plates were coated overnight at 4°C with Spike glycoprotein (Sino Biological) at 1 μg/mL in PBS. After washing, plates were blocked with PBS/1% BSA (Miltenyi Biotec) for 2 h at RT. Samples were diluted in blocking buffer at 0.5 AU/mL, added to the corresponding wells, and evaluated by quadruplicate. Samples were incubated for 2 h at RT. Two of the four wells were incubated with 2M guanidine HCl, and the other two with PBS for 15 min at RT. Guanidine HCl was selected as a chaotropic agent because it has been described to have the ability to disrupt antibody-antigen interactions.^35^ Plates were washed again, and the bound antibodies were detected using an HRP-conjugated goat anti-human IgG (1/20,000) (Jackson ImmunoResearch), which was incubated for 30 min at RT. Plates were revealed using OPD (Sigma Aldrich) and the enzymatic reaction was stopped with 2M of H_2_SO_4_ (Sigma Aldrich). Signal was evaluated as the OD at 492 nm with noise correction at 620 nm. Avidity index was calculated as the ratio between mean signal obtained with and without guanidine treatment.

#### Pseudovirus production and neutralization assay

HIV reporter and replication-incompetent pseudoviruses expressing either WH1, Delta, or Omicron Spike proteins and luciferase were produced. pNL4-3.Luc.R-.E−was obtained from the NIH AIDS Reagent Program. SARS-CoV-2.SctΔ19 was generated from the full protein sequence of SARS-CoV-2 Spike (GeneArt), including a deletion of 19 amino acids in at the end of the C-terminal. This sequence was human-codon optimized and inserted into pcDNA3.4-TOPO. Expi293F cells were transfected using ExpiFectamine293 reagent (Thermo Fisher Scientific) with both pNL4-3.Luc.R-.E− and SARS-CoV-2.SctΔ19 plasmids at a 24:1 ratio, respectively. Control pseudoviruses were obtained by replacing the Spike protein with a vesicular stomatitis virus G (VSV-G)-expressing plasmid. Supernatants were harvested 48 h after transfection, filtered at 0.45 μm, frozen, and titrated on HEK293T cells overexpressing wild type human ACE-2 (Integral Molecular).^34^^,^^36^ Neutralization assays were performed in duplicate. Briefly, in 96-well cell culture plates (Thermo Fisher Scientific), 200 TCID_50_ were preincubated with 3-fold serial dilutions (1/60–1/14,580) of heat-inactivated plasma samples for 1 h at 37°C. Then, 2 × 10^4^ HEK293T/hACE2 cells treated with DEAE-Dextran (Sigma-Aldrich) were added onto those wells. Luciferase levels were developed using Britelite Plus Luciferase reagent (PerkinElmer) 48 h later, and read using the Ensight Multimode Plate Reader. Neutralization ID_50_ titers (reciprocal 50% inhibitory dilution) were calculated using non-linear fit of transformed data in GraphPad Prism v8.0 (GraphPad Software).

#### IFN-γ ELISPOT

ELISpot was performed using the Human IFN-γ ELISpot kit (ALP) (Mabtech). ELISpot plates (Millipore) were coated with the anti-IFN-γ 1D1K antibody at 2 μg/mL. PBMCs were thawed and rested for 3 h at 37°C in RPMI-1640 media supplemented with 10% FBS, and 1% penicillin/streptomycin (R10) (Thermo Fisher Scientific). Anti-CD40 antibody was added (0.5 μg/mL, HB14, Miltenyi Biotec) to prevent CD40L internalization 15 min prior peptide stimulation.^28^ Anti-CD49days was also added as a costimulator (1 μg/mL, 9F10, Thermo Fisher Scientific). PBMCs were then stimulated for 16 h with either: (1) R10; (2) CytoStim (Miltenyi Biotec); or (3) Spike-S1 peptide pool (Miltenyi Biotec). Plates were washed and were firstly incubated for 2 h at RT with the biotinylated anti-human IFN-γ 7-B6-1 antibody (1 μg/mL), and then with streptavidin-ALP (1/1,000) for 1 h at RT. Last, wells were developed using BCIP/NBT-plus substrate (BioRad). Spots were enumerated using an ImmunoSpot reader (Cellular Technologies Limited). PBMCs from an unvaccinated SARS-CoV-2-negative donor, and from a BNT162b2-vaccinated individual were used as negative and positive controls, respectively. A positive response was considered if a sample showed five or more SARS-CoV-2-specific cells per 10^5^ PBMCs, and at least a 2-fold increase compared to w0.

#### Flow cytometry

PBMCs were stimulated with anti-CD40 (0.5 μg/mL, HB14, Miltenyi Biotec), anti-CD49days (1 μg/mL, 9F10, Thermo Fisher Scientific), and Spike-S1 peptides (Miltenyi Biotec) for 16 h at 37°C, as described above. Particularly, an anti-CD40 antibody was used to allow the detection of CD154 on the cell surface of antigen-specific cells by preventing the internalization of CD40L (CD154).^28^ Anti-CD49days antibody was utilized to costimulate T cells, as described in Waldrop et al.^37^ The following day, cells were incubated for 30 min at RT with live/dead fixable aqua (Thermo Fisher Scientific). Fcγ receptors were blocked using human truStain FcX (BioLegend) and incubate for 5 min before adding fluorochrome-conjugated antibodies. Cells were stained for 20 min at RT with: CD5-R718 (UCHCT2), CD8-APC-H7 (SK1), CD4-BV605 (RPA-T4), CD25-BV421 (2A3), CD14-V500 (M5E2), CD19-V500 (Hib19), CD154-BB700 (Trap-1), CD137-APC (4B4-1) from BD Biosciences, and OX40-PE-Cy7 (Ver-ACT35) and CD69-PE (FN50) from BioLegend. Samples were acquired on a BD LSRII, and analyzed using FlowJo software (Treestar).

### Quantification and statistical analysis

#### Statistical analysis

ELISA binding data, neutralizing antibody titers, and IFN-γ spot-forming cells per 10^5^ cells are shown as mean ± SD. Differences between PAD and HC groups were established using Kruskal-Wallis test corrected for multiple comparisons using Dunn’s test. Wilcoxon signed-rank test was used to identify significant differences elicited over time in one group. A significance threshold of 0.05 was used for each statistical test, and all p values reported were two-tailed. Dose-response neutralization curves were fit to a logistic equation by non-linear regression analysis. Statistical analysis was performed using GraphPad Prism v8.0.

## Data Availability

• All data reported in this article will be shared by the lead contact on request. • This article does not report original code. • Any additional information required to reanalyze the data reported in this article is available from the lead contact on request.
