# Supplementary material for: Kinetics of immune responses elicited after three mRNA COVID-19 vaccine doses in predominantly antibody-deficient individuals
Source: iScience. 2022 Oct 28;25(11):105455. doi: 10.1016/j.isci.2022.105455 (PMC9613776; doi:10.1016/j.isci.2022.105455)
Supplement: Document S1. Figures S1–S3 and Table S1 [file mmc1.pdf]

iScience, Volume *25*

Supplemental information

Kinetics of immune responses elicited after three mRNA COVID-19 vaccine doses in predominantly antibody-deficient individuals

Erola Ainsua-Enrich, Núria Pedreño-Lopez, Carmen Bracke, Carlos Ávila-Nieto, María Luisa Rodríguez de la Concepción, Edwards Pradenas, Benjamin Trinité, Silvia Marfil, Cristina Miranda, Sandra González, Ruth Toledo, Marta Font, Susana Benet, Tuixent Escribà, Esther Jimenez-Moyano, Ruth Peña, Samandhy Cedeño, Julia G. Prado, Beatriz Mothe, Christian Brander, Nuria Izquierdo-Useros, Julia Vergara-Alert, Joaquim Segalés, Marta Massanella, Rosa María Benitez, Alba Romero, Daniel Molina-Morant, Julià Blanco, Bonaventura Clotet, Lourdes Mateu, María Luisa Pedro-Botet, and Jorge Carrillo

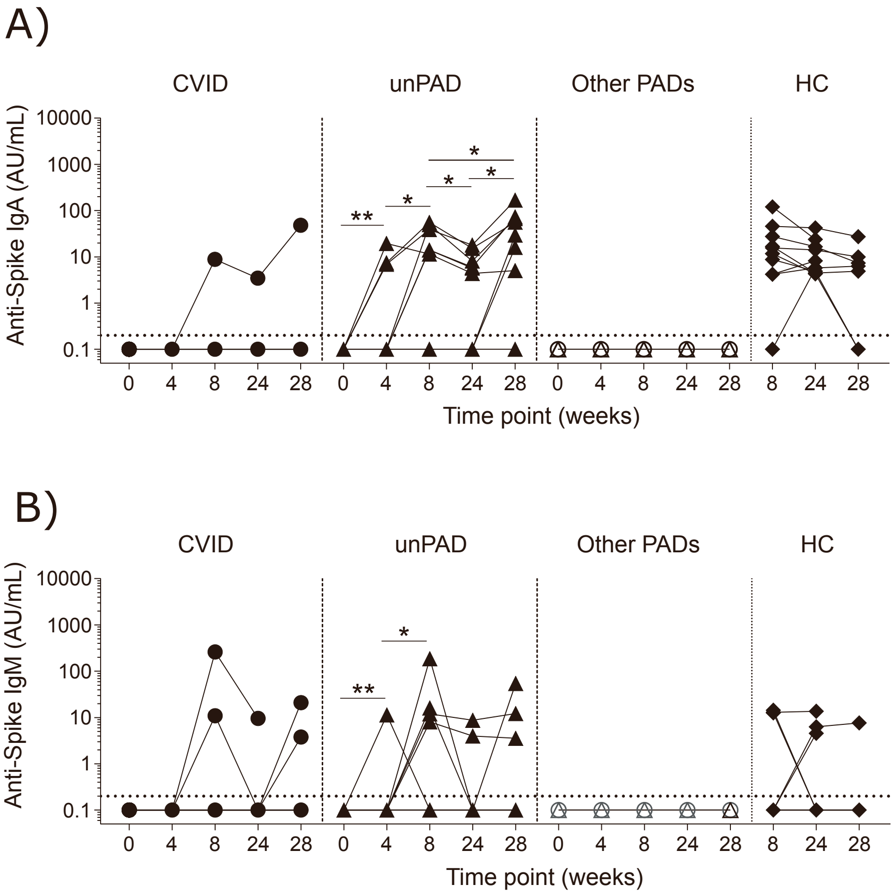

SUPPLEMENTAL FIG 1. Kinetics of Anti-Spike IgM and IgA responses in vaccinated PAD patients, related to Figure 1. Vaccine-induced IgA (A) and IgM (B) titers in CVID (n=12, black circles), unPAD (n=9, black triangles), other PADs (CID: n=1, open triangles and TID: n=1, open circles), and HC groups (n=10, black diamonds) after vaccination. Data were analyzed using Wilcoxon signed rank test * P < .05; ** P <.01.

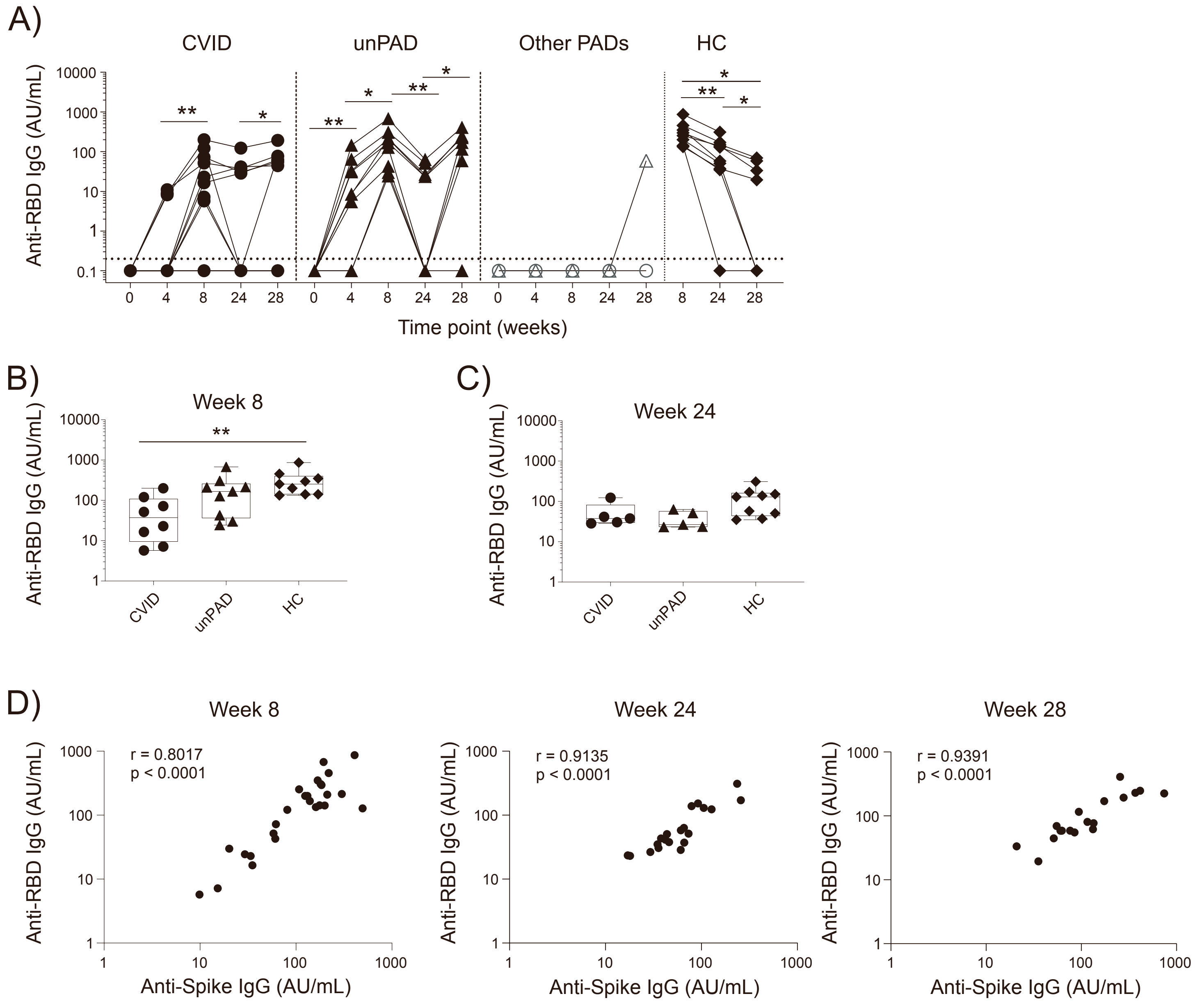

SUPPLEMENTAL FIG 2. Kinetics of RBD-specific IgG responses in vaccinated PAD patients, related to Figure 1. A) Levels of anti-RBD IgG antibodies were determined in CVID (n=12, black circles), unPAD (n=9, black triangles), other PAD (CID: n=1, open triangles and TID: n=1, open circles) and HC groups (n=10, black diamonds) after vaccination. Comparison of RBD-specific IgG levels among CVID, unPAD, and HC groups at w8 **(B)**, and w24 **(C)** after the first vaccine dose. **D)** Correlation between anti-RBD and anti-Spike IgG levels, which was determined using Spearman's rank correlation. Data in A was analyzed using Wilcoxon signed rank test. Data in B and C were analyzed using Dunn's Multiple Comparison Test. * P < .05; ** P < .01; ***P<.001.

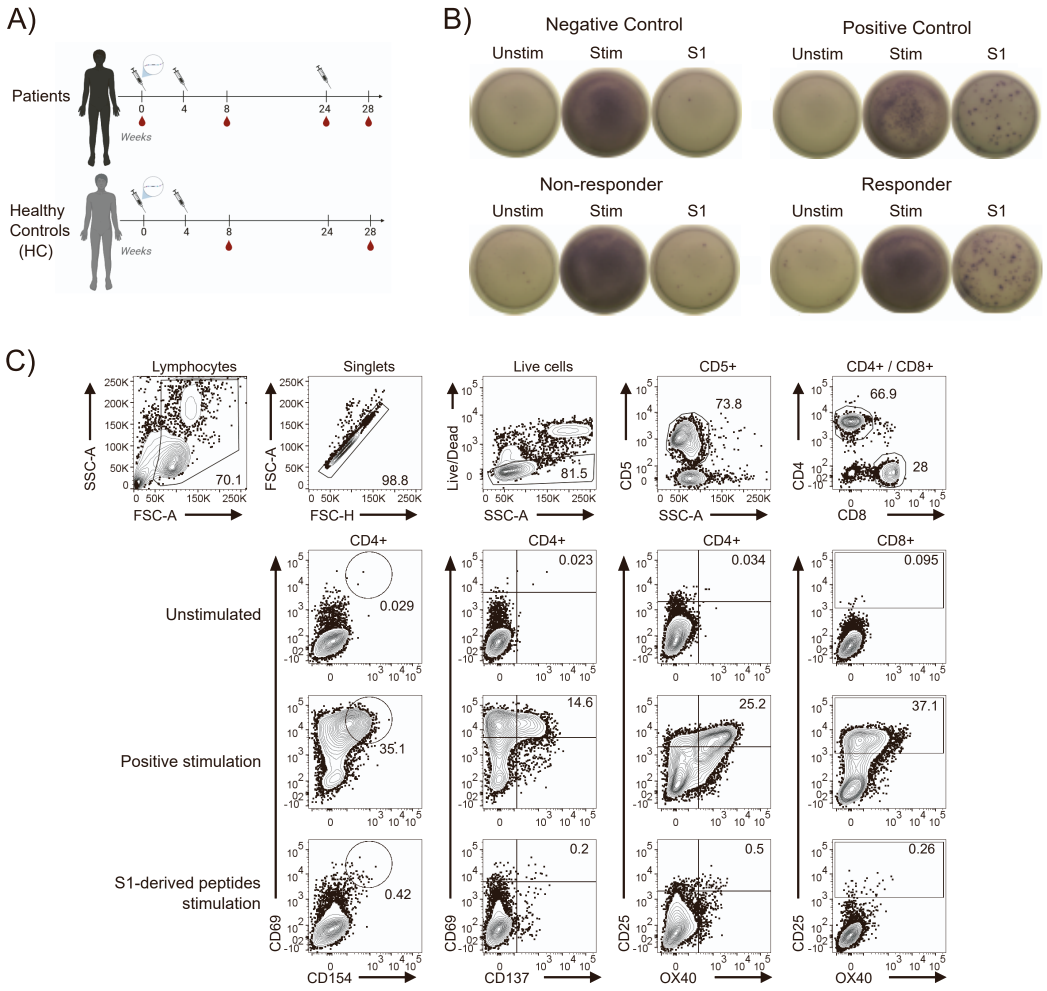

SUPPLEMENTAL FIG 3. Characterization of vaccine-induced cellular immune response in PAD patients, related to Figure 4, 5 and 6. A) Vaccine regimen timeline and sample collection. **B)** Representative examples of IFN-γ ELISpot. **C)** Gating strategy used to determine activation-induced markers in both CD8+ and CD4+ T cells after PBMC stimulation with S1-derived peptides for 16 hours: lymphocytes / live / CD5+ / CD4+ or CD8+ / CD69+CD154+; CD69+CD137+; CD25+OX40+. A representative analysis is shown using all three stimulation conditions: unstimulated (Unstim), positive control stimulated using cytostim (Stim, Miltenyi Biotec), and S1 peptides (S1, Miltenyi Biotec).

SUPPLEMENTAL Table 1. Vaccine-induced adverse effects in SARS-CoV-2-uninfected PAD patients, related to Table 1.

Adverse events	First dose	Second dose	Third dose
Local pain	19 (82.6%)	21 (91.3%)	19 (82.6%)
Local blush	2 (8.7%)	4 (17.4 %)	6 (26%)
Local inflammation	2 (8.7%)	5 (21.7%)	7 (30.4%)
Paresthesia	0 (0%)	1 (4.4%)	1 (4.4%)
Headache	5 (21.7%)	6 (26%)	7 (30.4%)
Shivers	3 (13%)	8 (34.8%)	10 (43.5%)
Arthromyalgia	2 (8.7%)	10 (43.5%)	9 (39.1%)
Asthenia	7 (30.4%)	11 (47.8%)	13 (56.6%)
Dizziness	0 (0%)	3 (13%)	3 (13%)
Syncope	0 (0%)	0 (0%)	0 (0%)
Nausea/vomiting	1 (4.4%)	2 (8.7%)	1 (4.4%)
Diarrhea	2 (8.7%)	1 (4.4%)	3 (13%)
Fever	1 (4.4%)	8 (34.8%)	9 (39.1%)
Local adenopathy	0 (0%)	0 (0%)	1 (4.4%)
Anaphylaxis	0 (0%)	0 (0%)	0 (0%)
Other adverse events	2 (8.7%)	4 (17.4%)	5 (21.7%)
Medical assistance	0 (0%)	1 (4.4%)	0 (0%)
Sick leave	0 (0%)	1 (4.4%)	0 (0%)
Days of sick leave	0	3	0
Hospitalization	0 (0%)	0 (0%)	0 (0%)
